# Simulations of Asymmetric Membranes Illustrate Cooperative Leaflet Coupling and Lipid Adaptability

**DOI:** 10.3389/fcell.2020.00575

**Published:** 2020-07-21

**Authors:** Madison Blumer, Sophia Harris, Mengzhe Li, Luis Martinez, Michael Untereiner, Peter N. Saeta, Timothy S. Carpenter, Helgi I. Ingólfsson, W. F. Drew Bennett

**Affiliations:** ^1^Harvey Mudd College, Claremont, CA, United States; ^2^Scripps College, Claremont, CA, United States; ^3^Claremont McKenna College, Claremont, CA, United States; ^4^Pomona College, Claremont, CA, United States; ^5^Biochemical and Biophysical Systems Group, Biosciences and Biotechnology Division, Lawrence Livermore National Laboratory, Livermore, CA, United States

**Keywords:** lipid membrane, membrane asymmetry, molecular dynamic simulations, cholesterol, multiscale modeling

## Abstract

Biological membranes are composed of lipid bilayers that are often asymmetric with regards to the lipid composition and/or aqueous solvent they separate. Studying lipid asymmetry both experimentally and computationally is challenging. Molecular dynamics simulations of lipid bilayers with asymmetry are difficult due to finite system sizes and time scales accessible to simulations. Due to the very slow flip-flop rate for phospholipids, one must first choose how many lipids are on each side of the bilayer, but the resulting bilayer may be unstable (or metastable) due to differing tensile and compressive forces between leaflets. Here we use molecular dynamics simulations to investigate a number of different asymmetric membrane systems, both with atomistic and coarse-grained models. Asymmetries studied include differences in number of lipids, lipid composition (unsaturated and saturated tails and different headgroups), and chemical gradients between the aqueous phases. Extensive analysis of the bilayers’ properties such as area per lipid, density, and lateral pressure profiles are used to characterize bilayer asymmetry. We also address how cholesterol (which flip-flops relatively quickly) influences membrane asymmetries. Our results show how each leaflet is influenced by the other and can mitigate the structural changes to the bilayer overall structure. Cholesterol can respond to changes in bilayer asymmetry to alleviate some of the effect on the bilayer structure, but that will alter its leaflet distribution, which in turn affects its chemical potential. Ionic imbalances are shown to have a modest change in bilayer structure, despite large changes in the electrostatic potential. Bilayer asymmetry can also induce a modest electrostatic potential across the membrane. Our results highlight the importance of membrane asymmetry on bilayer properties, the influence of lipid headgroups, tails and cholesterol on asymmetry, and the ability of lipids to adapt to different environments.

## Introduction

Lipid membranes separate organelles and cells from their exterior environments. Lipids can spontaneously assemble into a bilayer, which is the basic structure of cell membranes. These lipid bilayers function as a selective barrier to molecular entry/exit for the cell and serve as planar platforms for colocalizing various cell machinery. Because of their small scale, complexity and fluid phase behavior, it is difficult to study lipid bilayers directly, particularly *in vivo*. It has been long established that most cellular membranes have an asymmetric distribution of lipids between the inner and outer bilayer leaflets. Quantifying lipid compositions in either leaflet, transleaflet exchange (flip-flop), how cells create and maintain asymmetry, and the biological and physical effects of membrane asymmetry remain open research areas.

There are a number of ways that a membrane can exhibit asymmetry: different numbers of lipids, different types of lipids, asymmetric proteins, and by separating chemically different aqueous compartments. Removing lipids from one leaflet creates a stress in the membrane and can result in membrane curvature. Extreme examples are small unilamellar vesicles, which have high curvature and different numbers of lipids in either leaflet. In cells, regions of high curvature, such as membrane tubules, exosomes, and during vesicle fission/fusion, can results in lipid asymmetry (Callan-Jones et al., 2011; Skotland et al., 2017; Gruenberg, 2020). Cells tightly control the transbilayer distribution of lipids. Mammalian plasma membranes have phosphatidylcholine and sphingomyelin enriched in the outer leaflet and phosphatidylethanolamine and phosphatidylserine enriched in the inner leaflet (Van Meer et al., 2008; Lorent et al., 2020). The localization of cholesterol and rate of flip-flop remains debated (Lange and Steck, 2008; Mondal et al., 2009; Steck and Lange, 2018). Disruption of the membrane asymmetry with phosphatidylserine exposure on the outer leaflet is an important cell signal, for example during apoptosis (Van Meer et al., 2008). *In vivo* this is even more complex, because domains or lipid rafts can cause a heterogenous lipid distribution within the same leaflet. Considerable effort has been spent studying the interplay between lipid phase separation in one leaflet and asymmetric distribution of lipids across the membrane (Collins and Keller, 2008; Perlmutter and Sachs, 2011). A main question is if and how ordered domains enriched in cholesterol and sphingomyelin in the external leaflet can induce domains in the inner leaflet. Complexity is furthered by the active transport of lipids across membranes by transmembrane proteins. Additionally, cells tightly control the ion and osmotic gradients across the membrane with transmembrane protein channels and transporters. Key examples of how cells actively use the electrostatic potential difference are nerve cell propagation and energy production. The situation is even more complex, because the activity of the channels and transporters that create and maintain these asymmetries are in turn affected by their local lipid and ionic environment. Teasing out the interplay between the physicochemical effects of asymmetry and biological processes is ongoing.

Advances in computer simulations of lipid bilayers have made simulating complex and more biologically relevant membranes possible. This is exemplified by recent simulations on a realistic plasma membrane (Marrink et al., 2019), a neuronal membrane (Ingólfsson et al., 2017), bacterial membranes (Khalid et al., 2015), and entire organelles, such as a chromatophore vesicle (Singharoy et al., 2019). While at the outset it seems trivial for one to set up an asymmetric membrane *in silico*, as one can simply place lipids on one side or the other, there are problems that can create artifacts (Park et al., 2015; Huber et al., 2019). Finite system sizes means small bilayer patches are necessary, which can influence asymmetry effects, such as membrane bending. Simulation times that are very short compared to slow lipid movements, such as phospholipid flip-flop (McNamee and McConnell, 1973; Tieleman and Marrink, 2006; Bennett et al., 2009b; Marquardt et al., 2017), can influence simulation results. Including transmembrane proteins that have asymmetric shape further complicates this problem. Therefore, one *can* simulate nearly any asymmetry one chooses, but deciding what is relevant is a more difficult problem. Using the Martini coarse-grained (CG) model (Marrink et al., 2007), it was shown that even completely removing one leaflet (i.e., infinite asymmetry) was possible in relatively short and small simulations due to the strong stabilizing effect of periodic boundary conditions (Esteban-Martín et al., 2009), and slow time scale for phospholipid flip-flop. Another example showed that a CG simulation of a POPC membrane with 15 mol% difference in the number of lipids between leaflets was metastable, and applying a small pressure to break the metastability resulted in a membrane bud forming (Siggel et al., 2019). A transmembrane scramblase protein was then shown to alleviate the asymmetry.

To date most molecular dynamics (MD) simulations of lipid bilayers have been symmetric in the composition of their two leaflets. Many simulations of complex asymmetric bilayers have chosen the composition based on matching the average area per lipid (APL) of symmetric bilayers that represent each of the different desired leaflets (Huber et al., 2019). Recent work by Doktorova and Weinstein (2018) suggest that an asymmetric membrane should be constructed not just so the overall membrane tension is zero but so the tension in each leaflet is zero. Subsequently Hossein and Deserno (2020) showed that differential stress can still exist within membranes with non-zero tension, suggesting a detailed balancing of compositional and lateral stress is needed for balancing the physical effects of asymmetry. There has also been a number of simulations investigating the role of ion and lipid gradients on the transmembrane electrostatic potential (Gurtovenko and Vattulainen, 2007; Gurtovenko and Lyulina, 2014). Large ion imbalances lead to membrane electroporation, where a water filled pore forms across the bilayer, which has also been studied extensively with simulations (Gurtovenko and Vattulainen, 2005; Böckmann et al., 2008; Gurtovenko et al., 2010). MD simulations have also looked at cholesterol in lipid domains and have provided physical insight into domain registration for asymmetric membranes (Perlmutter and Sachs, 2011; Tian et al., 2016). Simulations have shown how liquid-ordered domains on one leaflet can promote more order in the opposite leaflet (Perlmutter and Sachs, 2011; Tian et al., 2016).

We have conducted a systematic investigation of different types of membranes simulated with multiscale simulation protocols. This work provides detailed physical insights in membrane asymmetry with biological implications as well as interesting technical details for the simulation community. MD simulations using atomistic and CG models of lipid bilayer asymmetry are presented and the bilayers’ properties are analyzed and compared. We address a number of asymmetric membrane scenarios: a gradual change in asymmetry, different numbers of lipids, different types of lipid, and ion imbalances, with and without cholesterol. We calculate lipid properties and bilayer properties, including lipid density profiles, electrostatic potentials, lipid order parameters, APL, cholesterol flip-flop and lateral pressure profiles. These results show how changes in lipid asymmetry can have modest to no effect on some membrane properties but rather large changes for other properties, emphasizing when more detailed attention to asymmetry is necessary. By comparing atomistic and CG models, we can assess model and chemical differences and inform future work. Simulations with cholesterol illustrate how lipids that can flip-flop readily can alter the asymmetry and influence the resulting membrane properties.

## Materials and Methods

### Large CG Lipid Membrane Simulations and Setup

We set up the larger CG lipid bilayers with varying degrees of asymmetry using the Martini tool *insane* (Wassenaar et al., 2015). These where simulated at the CG resolution only, contained up to 222 lipids in each leaflet and were run with an asymmetric DPSM (d18:1/18:0-sphingomyelin), POPC (16:0/18:1-PC), and PAPE (16:0/20:4-phosphatidylethanolamine) lipid mixture with a 40/40/20 ratio in the upper leaflet and 10/40/50 in the lower leaflet. Simulations were done with and without a high 50 mol% cholesterol content with lipid count asymmetry ranging from 0 to 35 lipids from the lower leaflet. All simulations were setup with 150 mM NaCl (∼75 beads each) and >15 CG water beads per lipid (∼6700 beads), corresponding to >60 waters per lipid.

### Small CG and AA Lipid Membrane Simulation Setup

Systems with up to 64 lipids in each leaflet were setup with DPPC (16:0-PC) and DIPC (18:2-PC) in both leaflets, with 0, 5, or 10 lipids removed from one leaflet (e.g., DPPC_5). Asymmetric membranes with DPPC in one leaflet and DIPC in the other were simulated [DIPC(upper)/DPPC(lower)], and with 5 and 10 lipids removed from the DIPC leaflet (e.g., DPPC_0_DIPC_5). Each system was simulated at the atomistic and CG resolution and with and without ∼30 mol% cholesterol (relative to the upper layer) placed in each leaflet at the start of the simulation (replacing the PC lipid to maintain 64 lipids). Simulations were done with an asymmetric bilayer with DPPE (16:0-PE) in the upper leaflet and DPPC in the lower leaflet (headgroup asymmetry), both with 64 lipids. Additionally, at the CG resolution a larger range of asymmetries for the DIPC(upper)/DPPC(lower) were simulated (see Supplementary Figure S3). Each system contained 150 mM NaCl (∼22 beads each) and ∼2000 CG water beads. We set up double bilayer systems by replicating a single bilayer twice in the z-dimension. For asymmetric bilayers we also flipped the bilayer leaflets, so that the upper and lower are on opposite sides for the two bilayers. To create ion imbalances, we simply changed one sodium to a chloride in one water bath and chloride to sodium in the other, leading to a 4e imbalance.

### Martini Simulations

We used the Martini CG force field v2.0 (Marrink et al., 2007) and a recent (unpublished) version of cholesterol that updated the shape of the original cholesterol model (Marrink et al., 2007) similar to the updated cholesterol model described in Melo et al. (2015) but without using virtual-sites. A 20 fs time step was used with the leap-frog algorithm and the new-rf Martini simulation parameters (De Jong et al., 2016). Each of the 64 lipids per leaflet simulations was simulated for 2 μs at 333 K and the 222 lipids per leaflet simulations for 10 μs at 320 K, both using the velocity rescale thermostat (Bussi et al., 2007) with a 12 ps time constant. The systems with DPPE were simulated at 340 K to be above the melting temperature for DPPE. Semi-isotropic pressure coupling was used with Berendsen barostat (Berendsen et al., 1984) with 1 bar pressure, a compressibility of 3 × 10^–4^ bar^–1^, and a relaxation time of 12 ps. Electrostatics were computed using the reaction-field method, with a dielectric constant of 15 within a 1.1 nm cutoff and infinite beyond the cutoff.

### Atomistic Simulations

CHARMM36 lipids (Klauda et al., 2010) with CHARMM specific (Durell et al., 1994; Neria et al., 1996) TIP3P water (Jorgensen et al., 1983) were simulated with GROMACS v5.1.4 (Van Der Spoel et al., 2005; Abraham et al., 2015). The starting structure was taken from the 200 ns frame of the Martini CG model and back mapped to CHARMM36 using the Backward method (Wassenaar et al., 2014). Simulations were then continued using a 2 fs time step, with four replicates of 250 ns, with the final 200 ns from each used for analysis. Lennard-Jones interactions were cut-off after 1.2 nm, with a force-switch-function from 1.0 to 1.2 nm. Long-range electrostatic interactions were calculated with the particle mesh Ewald method (Darden et al., 1993; Essmann et al., 1995). Temperature was maintained at 333 K using the Nose-Hoover thermostat (Martyna et al., 1992) with a time constant of 1 ps. Semi-isotropic pressure coupling was used to maintain a constant pressure of 1 bar lateral and normal to the plane of the bilayer. The Parrinello-Rahman barostat (Parrinello and Rahman, 1981) was used with a compressibility of 4.5 × 10^–5^ and a coupling constant of 5 ps. Water was constrained with the SETTLE method (Miyamoto and Kollman, 1992), and hydrogens on lipids with P-LINCS (Hess et al., 1997; Hess, 2008).

### Simulation Analyses

After the simulations we used a variety of computational methods to analyze the lipid membrane systems. The different analysis methods use a combination of GROMACS tools, custom Python scripts using MDAnalysis (Michaud-Agrawal et al., 2011; Melcr et al., 2018; Antila et al., 2019). as well as the FATSLiM program (Buchoux, 2016) for APL calculations using Voronoi tessellation and membrane thickness calculations. The atomistic lipid tail order parameter was calculated using:

$$S_{\text{CH}}=<3\,c\,o\,s^{2}\,\theta -1>/2$$

where θ is the angle between the C-H bond and the normal to bilayer. This was calculated using an analysis script in MDAnalysis (Michaud-Agrawal et al., 2011; Melcr et al., 2018; Antila et al., 2019). Density and electrostatic profiles were calculated with an in-house script using MDAnalysis (Michaud-Agrawal et al., 2011). The electrostatic profile was determined using the method of Sachs et al. (2004), where the partial charge distribution across the membrane is integrated twice to give the electrostatic potential. For electrostatic potentials with asymmetry, we used a double bilayer set-up, allowing the two water chambers to have different ion concentrations.

**FIGURE 1:**
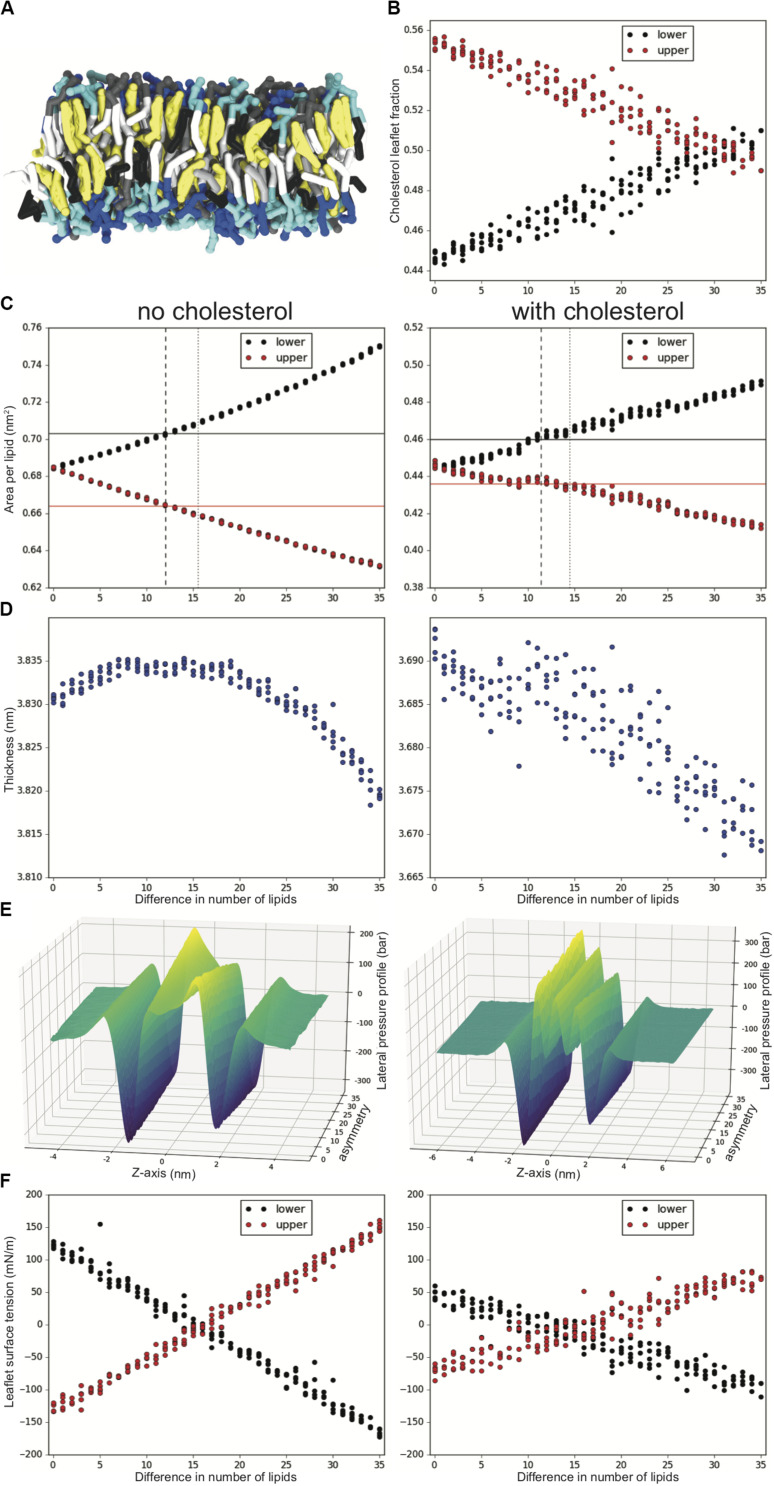
Tuning lipid number asymmetry for an asymmetric DPSM/POPC/PAPE 40:40:20 upper and 10:40:50 lower mixture, with and without 50% cholesterol. **(A)** Snapshot showing a sideview of a simulation with cholesterol and a difference in number asymmetry of 20 lipids. Tails are colored white, gray and black for 0, 1, and 4 double bonds, cholesterol is yellow and the headgroups are gray, blue and cyan for SM, PC and PE, respectively. **(B)** Cholesterol fraction in the upper and lower leaflet of each system. **(C)** Upper and lower leaflet APL for increased lipid number asymmetry. APL for symmetric upper (red) and symmetric lower (black) are shown in solid lines. Suggested number asymmetry value for this mixture from ratio of symmetric simulations and minimal leaflet ST are shown in dashed and dotted lines, respectively. **(D)** Bilayer thickness. **(E)** LPPs averaged over repeat simulations and outliers removed (Supplementary Figure S1). **(F)** Upper and lower leaflet ST. All analysis is averaged over the last 2 ms of the 10 ms simulations and there are 4–5 repeats for each lipid number asymmetry value.

The lateral pressure profile (LPP) was calculated using a modified version of GROMACS (Vanegas et al., 2014; Torres-Sánchez et al., 2015). The local stress is calculated by post-processing the trajectory file which returns a local stress file for the simulation averaged over the user-selected time range. We used the Goetz-Loetsky force decomposition and a grid spacing of 0.1 nm for atomistic and 0.15 nm for CG simulations. The local stress tensor (σ) is extracted from the binary file using the program tensortools (Vanegas et al., 2014; Torres-Sánchez et al., 2015), the LPP estimated as (σ_xx_ + σ_yy_)/2 and the leaflet surface tension (ST) for the upper and lower leaflet as the integral from the bilayer center of mass to the box end on either side. Note, for asymmetric bilayers the center of mass is not the exact center of the integrated LPP (where either leaflet has equal amplitude but opposite sign) but for the mixture simulated in Figure 1 the difference was within error of the LPP calculations see upper/lower symmetry in ST (Figure 1F).

Cholesterol exhibits the unique ability among the lipids used in this paper to flip from one leaflet to another over the course of a simulation. To investigate the effect of cholesterol mobility on asymmetry, we calculated the number of cholesterol molecules in each leaflet during the CG simulations. We considered cholesterols belonging to each leaflet if the ROH beads were >1.2 nm from the leaflet as defined by MDAnalysis leaflet finder using the non-flip-flopping lipids PO4 beads.

The combined analysis tools and a pipeline for setting up CG simulations is available, as is, on GitHub^1^.

## Results

### CG Membrane Asymmetry

To explore the desired lipid number asymmetry for complex asymmetrical bilayers we systematically simulated the asymmetric DPSM/POPC/PAPE mixture with 40:40:20 lipid ratio in the upper and 10:40:50 ratio in the lower leaflet, both with and without added 50 mol% cholesterol (Figure 1). In this mixture the upper leaflet has significantly more lipids with saturated tails (DPSM) and less with high level of unsaturation (PAPE) compared to the lower leaflet. The upper leaflet should therefore want to have a smaller APL than the lower leaflet. In a periodic system with a fixed number of lipids this would translate into a lipid number asymmetry with fewer lipids on the lower leaflet than the upper one. To determine what the number asymmetry should be, we systematically reduced the number of lipids in the lower leaflet, simulating 4–5 repeats at each asymmetry value ranging from 0 to 35 random lipids removed from the lower leaflet (reaching a number asymmetry of ∼16%). For the cholesterol-containing systems, both leaflets start with an equal amount of cholesterol, which then flip-flops between the leaflets. Figure 1B shows the cholesterol fraction in the upper and lower leaflets average over the last 2 ms (∼5% of the cholesterol is transitioning or resides in the bilayer middle and is excluded from this analysis). There is a net drive to move cholesterol from the lower leaflet into the upper leaflet, until a leaflet number asymmetry of 31 (14%) where the drive reverses.

Figure 1C shows the APL for systems with (right) and without (left) cholesterol as the number asymmetry increases. The APL increases for the lower leaflet and decreases for the upper leaflet in both systems as lipids are removed from the lower leaflet. The increase in the lower leaflet APL is ∼2% higher in amplitude than the decrease in the upper leaflet APL. For the same systems the bilayer thickness changes very little (∼0.02 nm) with increased asymmetry (Figure 1D, note the y-axes narrow range). With enough simulations the trend in thickness change can be picked up showing a reduced bilayer thickness with fewer lipids in both system types, with an interesting initial increase in thickness in the systems without cholesterol.

Figure 1E shows the LPP along the z-axis with increased number asymmetry, where each profile is the average of the 4–5 repeated simulations with a few outliers removed (Supplementary Figures S1, S2). For the systems without cholesterol in the upper leaflet (on the left of the LPP plots), the LPP starts with a deeper minimum and a shallower maximum which then reverses as the number asymmetry increases. This is the opposite for the lower leaflet (right side of the LPP plots). For the cholesterol containing systems the trend is less clear due to the lower relative change in the LPP profile and more complex shape. With increased number asymmetry the minimum for the upper leaflet (left side of the LPP plots) reduces in amplitude as well as the central maximum and the opposite holds for the maximum of the lower leaflet (right side of the LPP plots). Figure 1F quantifies the ST for each leaflet in all the systems (the integral of the LPP to the bilayer center). Both with and without cholesterol the amplitude and the ST difference between the leaflets reduces as the number asymmetry increases, and then flips sign and increases again. The amplitude in the cholesterol-containing systems is notably smaller in spite of the high range of the LPPs. The zero ST is reached at an asymmetry of 15–16 without cholesterol and 14–15 with cholesterol (Figure 1C dashed lines). For comparison asymmetry estimates from the APL ratio of symmetric upper and symmetric lower simulations give 12.1 (without cholesterol) and 11.4 (with cholesterol) (Figure 1C dotted lines).

**FIGURE 2:**
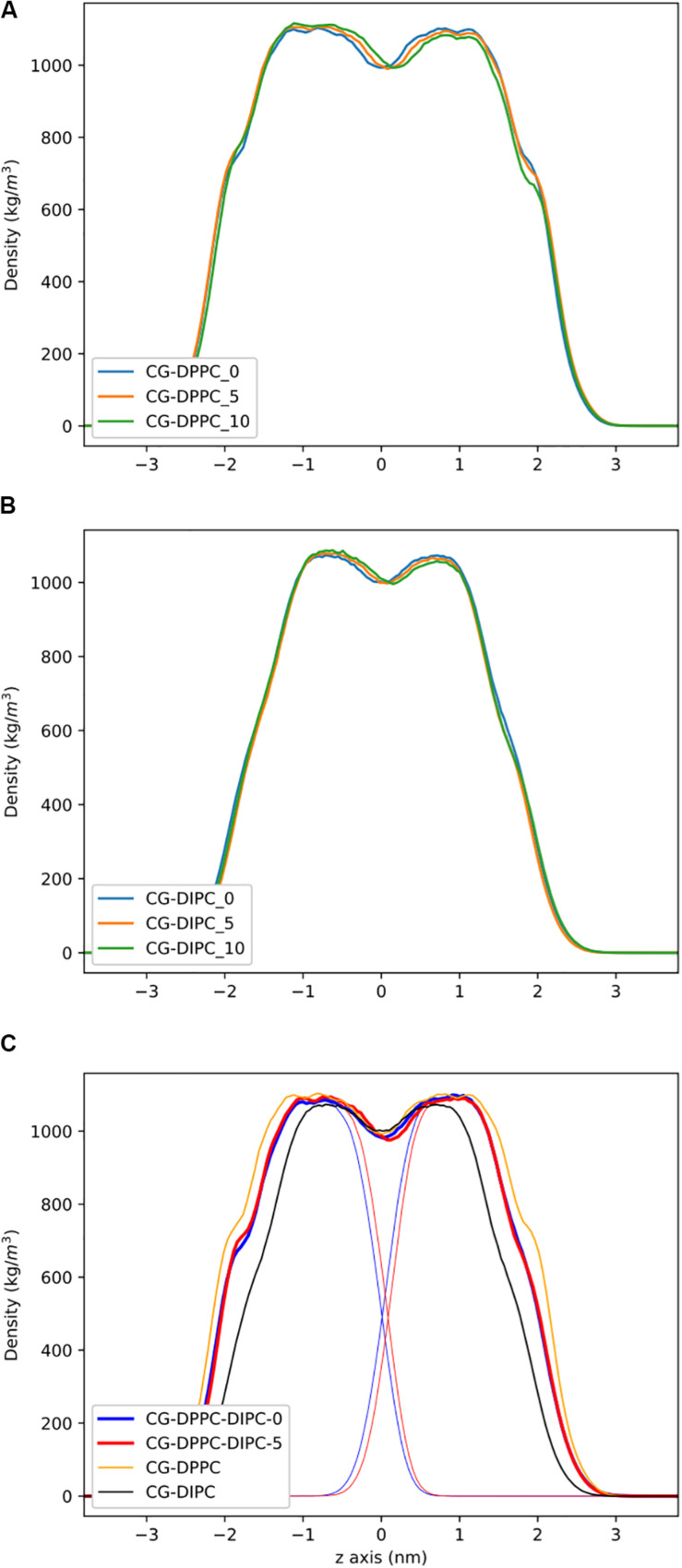
Partial density profiles for the CG lipid bilayers without cholesterol. **(A)** Profiles for DPPC. **(B)** Partial densities for DIPC. **(C)** DIPC(upper)/ DPPC(lower) bilayers, with thin lines showing the individual DIPC or DPPC densities.

To address the local effects of leaflet asymmetry in more detail, we compared atomistic and CG simulations of small bilayer patches composed of pure DPPC and pure DIPC. Figure 2 shows the density plots for the different CG bilayers. For the pure bilayers, a ∼8% (5 lipids) and ∼15% (10 lipids) difference in composition had a negligible effect on the lipid bilayer density. Particularly for the DIPC bilayer, the profiles are nearly identical. We then simulated a mixed bilayer with one leaflet DPPC and the other DIPC. The DIPC(upper)/DPPC(lower) bilayers had substantial changes, with a density in between the pure DPPC and DIPC density profiles (Figure 2C). Both leaflets respond in opposite directions, indicating that the DPPC leaflets became more fluid and the DIPC leaflets more structured. Similar to the pure bilayers, changing the number of lipids in the mixed bilayers (removing 5 DIPC), also had little effect on the density.

Table 1 lists the APL for each of the lipids in either the upper or lower leaflet. As expected, the leaflets with fewer lipids have a higher APL, and the ones with more lipids have a lower APL, compared to the pure bilayers. The mixed DIPC(upper)/DPPC(lower) bilayer has an APL in between the pure DPPC and DIPC membranes, as expected from the density profile. Supplementary Figure S3 plots the APL for the DIPC(upper)/DPPC(lower) bilayers as a function of the number of lipids removed from the DIPC(upper) leaflet. For reference, we also plot the APL for each type of lipid in pure symmetric bilayers (horizontal lines). The vertical line indicates the suggested asymmetry from symmetrical upper/lower leaflet simulations N(upper) = N(lower) × apl (lower)/apl(upper), which gives 51.8, or ∼12 fewer lipids for the DIPC(upper) leaflet.

**TABLE 1:** Area per phospholipid for the CG lipid bilayers.

**Name**	**Mol% cholesterol**	**DPPC (lower)**	**DPPC (upper)**	**DIPC (lower)**	**DIPC (upper)**
DPPC_0	0	0.644 (0.001)	0.644 (0.001)		
DPPC_5	0	0.620 (0.002)	0.672 (0.002)		
DPPC_10	0	0.605 (0.001)	0.715 (0.001)		
DIPC_0	0			0.795 (0.001)	0.795 (0.001)
DIPC_5	0			0.768 (0.001)	0.831 (0.001)
DIPC_10	0			0.742 (0.001)	0.878 (0.001)
DPPC_DIPC_0	0	0.702 (0.002)			0.702 (0.002)
DPPC_DIPC_5	0	0.668 (0.001)			0.725 (0.001)
DPPC_DIPC_10	0	0.638 (0.001)			0.755 (0.001)
DPPC_0	30	0.522 (0.001)	0.525 (0.0036)		
DPPC_5	30	0.512 (0.004)	0.521 (0.0073)		
DPPC_10	30	0.512 (0.004)	0.523 (0.0093)		
DIPC_0	30			0.662 (0.002)	0.661 (0.003)
DIPC_5	30			0.649 (0.004)	0.665 (0.005)
DIPC_10	30			0.633 (0.003)	0.673 (0.006)
DPPC_DIPC_0	30	0.494 (0.003)			0.679 (0.004)
DPPC_DIPC_5	30	0.488 (0.003)			0.696 (0.007)

**Error is shown in parentheses and units are nm^2^.**

We tested the effect of adding 30 mol% cholesterol to each of the bilayers. Cholesterol is able to flip-flop on a time scale accessible to CG simulations. This means that cholesterol is able to equilibrate between leaflets and affect the asymmetric bilayers structure. Supplementary Figure S4 shows how the number of cholesterol molecules changes during the CG simulations. Supplementary Figure S4A shows relatively slow exchange of cholesterol in the saturated DPPC bilayer and very fast exchange in the DIPC bilayer. For the asymmetric DIPC(upper)/DPPC(lower) bilayer, the cholesterol molecules concentrate in the DPPC leaflet, depleting the DIPC leaflet. These results match expectations from numerous previous studies on cholesterol flip-flop and chemical potential (Bennett et al., 2009a; Jo et al., 2010; Javanainen and Martinez-Seara, 2019). We note that the final structure at 200 ns was used to start the atomistic simulations. For the atomistic saturated DPPC bilayers, the cholesterol does not undergo flip-flop over the 1 μs of simulation, and rarely for the DIPC bilayer (Supplementary Figure S5). For the DPPC_0 system, we know that the cholesterol distribution must be equal, so we picked a frame (175 ns) that has an equal distribution of cholesterol, as opposed to the frame at 200 ns, which has an asymmetric distribution of cholesterol. For the asymmetric membranes, it is not clear that we have the correct distribution of cholesterol, because its chemical potential might be different in the two leaflets. It is not clear that the chemical potential difference between the CG and AA model would be the same, so we chose the frame at the end of the simulation. Future work could incorporate free energy calculations and/or simulate an ensemble of CG frames for AA conversion.

**FIGURE 3:**
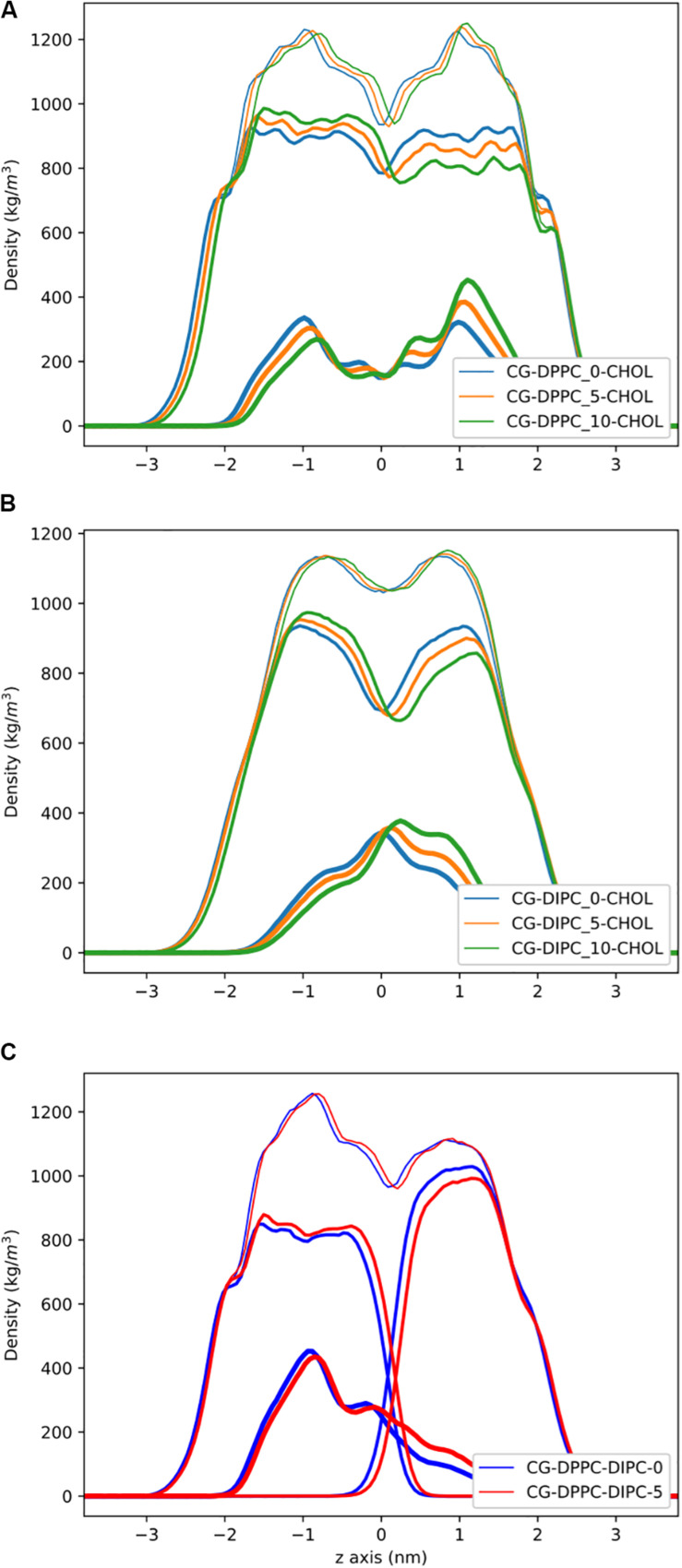
Partial density profiles for the CG lipid bilayers with 30 mol% cholesterol. **(A)** Densities for DPPC bilayers. **(B)** Partial densities for DIPC. **(C)** The DIPC(upper)/DPPC(lower) bilayer. Thin lines are for the whole bilayer, medium lines are the PC lipids, and cholesterol is thick lines.

Figure 3 shows the partial density profiles for the CG bilayers with 30 mol% cholesterol. These profiles show more substantial differences compared to the pure PC bilayers, but the same trends as bilayers without cholesterol. Notably, the DIPC and the DPPC densities have substantial differences compared to the symmetric bilayers, as well as cholesterol, but the overall bilayer density is similar for the asymmetric bilayers. Figure 3C illustrates how cholesterol is concentrated in the DPPC leaflet in the DIPC(upper)/DPPC(lower) bilayer. The APL for each CG bilayer are listed in Table 1 and show very modest changes with respect to the symmetric bilayers. Overall, the APL is much lower than the bilayers without cholesterol, due to cholesterols condensing effect.

**FIGURE 4:**
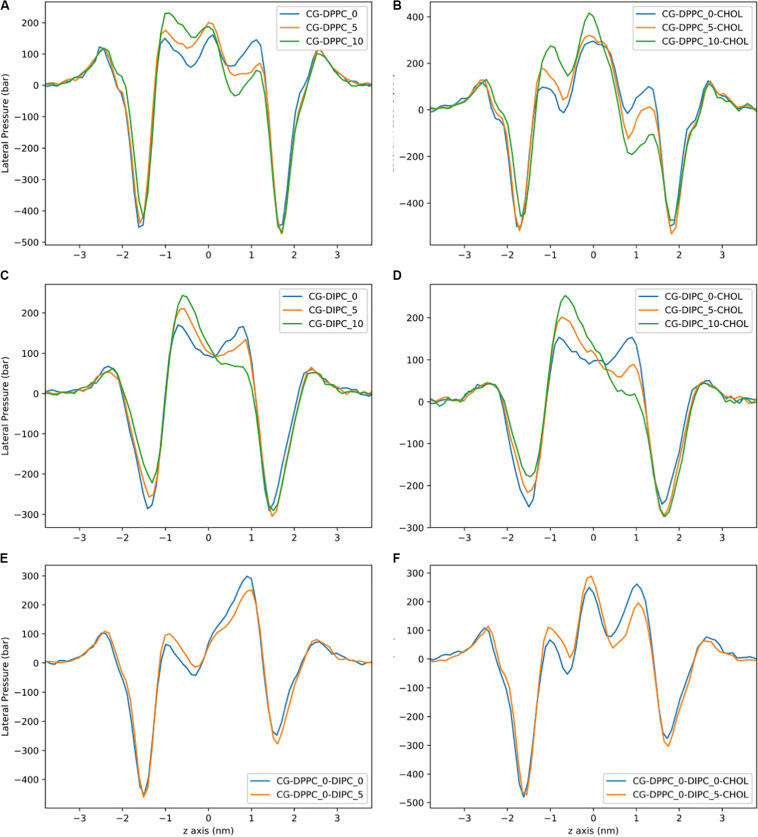
Lateral pressure profiles for CG bilayers with asymmetric number of lipids between leaflets. **(A)** DPPC. **(B)** DPPC:CHOL. **(C)** DIPC. **(D)** DIPC:CHOL. **(E)** DIPC(upper)/DPPC(lower). **(F)** DIPC(upper)/DPPC(lower):CHOL.

Lateral pressure profiles are an important property of lipid membranes, as they show the stress/pressure at each slice through the lipid membrane. Figure 4 shows the LPPs for the CG model with pure DPPC, pure DIPC, and both with 30 mol% cholesterol. For the pure bilayers, there is a large negative peak at the headgroup region and a shallow peak at the center of the membrane. Removing lipids from one leaflet of the DPPC bilayers has little effect on the height of the lateral pressure peak and trough (Figure 4A). There is a shift in the LPPS in the region corresponding to the middle of the lipid tails. A similar trend is observed for the polyunsaturated DIPC bilayers, although a slight decrease in pressure at the headgroup is also observed (Figure 4B). The magnitude of the pressures for the unsaturated DIPC bilayers are substantially lower than the saturated DPPC bilayers. Compared to the pure DPPC bilayers, LPPs for DPPC with 30 mol% cholesterol have a similar trend when removing lipids, but a much lower trough in pressure at the bilayer center (Figure 4C). The LPPs for the DIPC bilayers with and without cholesterol are very similar (Figure 4D compared to Figure 4B).

### AA Membrane Asymmetry

**FIGURE 5:**
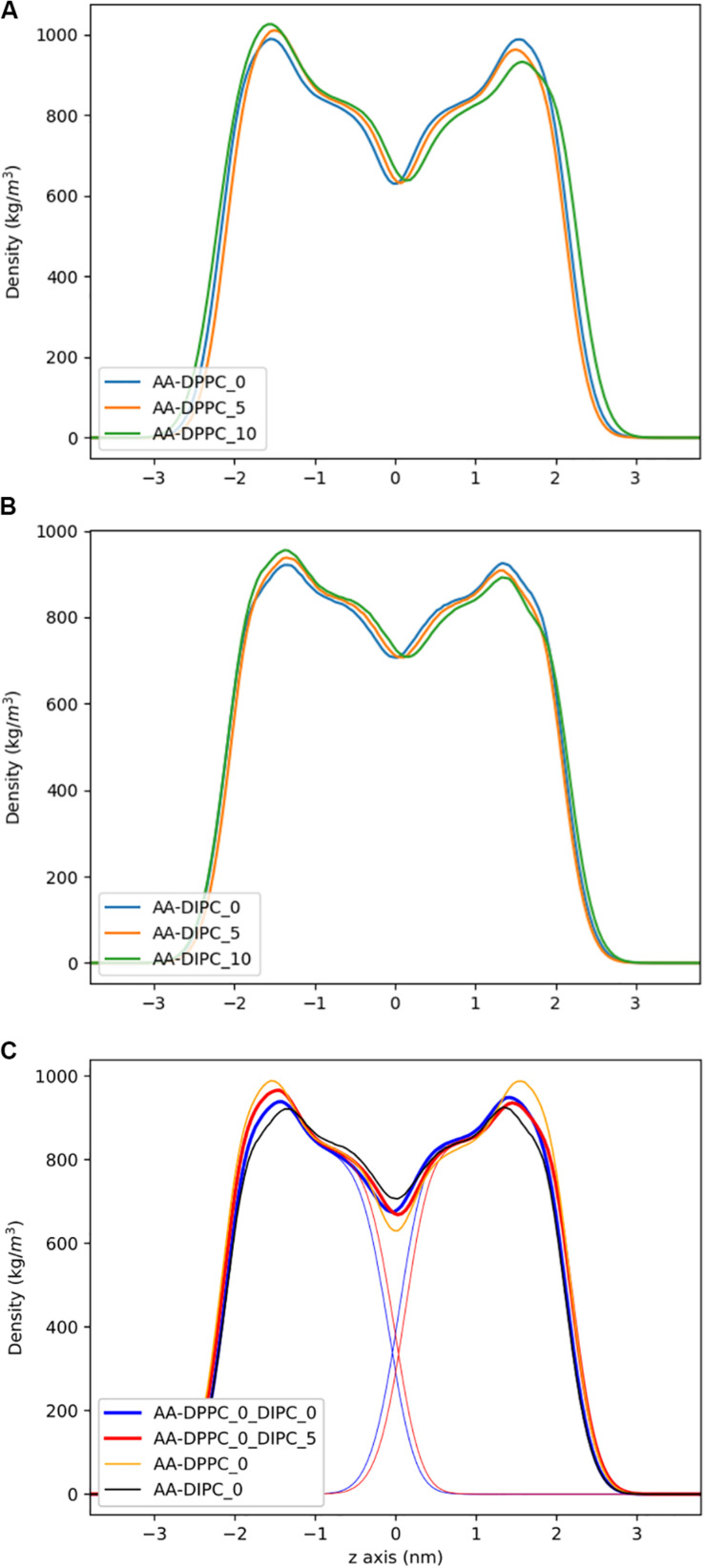
Partial density profiles for the AA bilayers without cholesterol. **(A)** DPPC density profiles. **(B)** DIPC density profiles. **(C)** Asymmetric bilayers with DPPC in one leaflet (lower) and DIPC in the other (upper). Thin lines are for densities for DPPC or DIPC lipids separately.

We tested the effect of membrane asymmetry on the structure of atomistic lipid membranes by simulating bilayers with the CHARMM36 lipids (AA). For each system, after 200 ns of CG simulation, the membrane structure was used to back map to an atomistic bilayer, using the Backward method (Wassenaar et al., 2014), and the AA simulation started. Figure 5 shows the resulting partial density profiles for the phospholipids in the membranes. These profiles show that without cholesterol the bilayer’s partial density is modified slightly by relatively large changes in the number of lipids in each leaflet. As expected, the leaflet with fewer lipids resulted in a slightly lower density, and the one with more resulted in a larger density compared to the symmetric bilayers. The differences in density are more pronounced than the CG density differences (Figure 2).

The APL was also calculated for each AA lipid bilayer (Table 2). The results show a trend expected from the shift in density profiles. By removing lipids in one leaflet, the opposite leaflet compensates, so the APL increases in the leaflet with fewer lipids and decreases in the leaflet with more lipids, compared to the pure bilayer. The DIPC(upper)/DPPC(lower) membrane has an APL in between the pure DPPC and pure DIPC areas per lipid.

**TABLE 2:** Area per phospholipid for the AA bilayers.

**Name**	**Mol% cholesterol**	**DPPC (lower)**	**DPPC (upper)**	**DIPC (lower)**	**DIPC (upper)**
DPPC_0	0	0.623 (0.003)	0.623 (0.002)		
DPPC_5	0	0.602 (0.001)	0.651 (0.001)		
DPPC_10	0	0.583 (0.001)	0.687 (0.001)		
DIPC_0	0			0.721 (0.001)	0.721 (0.001)
DIPC_5	0			0.696 (0.001)	0.753 (0.001)
DIPC_10	0			0.667 (0.002)	0.797 (0.002)
DPPC_DIPC_0	0	0.678 (0.001)			0.678 (0.001)
DPPC_DIPC_5	0	0.646 (0.001)			0.699 (0.001)
DPPC_0	30	0.558 (0.001)	0.529 (0.001)		
DPPC_5	30	0.550 (0.002)	0.534 (0.001)		
DPPC_10	30	0.518 (0.001)	0.561 (0.002)		
DIPC_0	30			0.641 (0.007)	0.632 (0.003)
DIPC_5	30			0.621 (0.006)	0.642 (0.002)
DIPC_10	30			0.620 (0.004)	0.651 (0.024)
DPPC_DIPC_0	30	0.524 (0.003)			0.677 (0.006)
DPPC_DIPC_5	30	0.522 (0.003)			0.675 (0.009)

**Error is shown in parentheses and units are nm^2^.**

**FIGURE 6:**
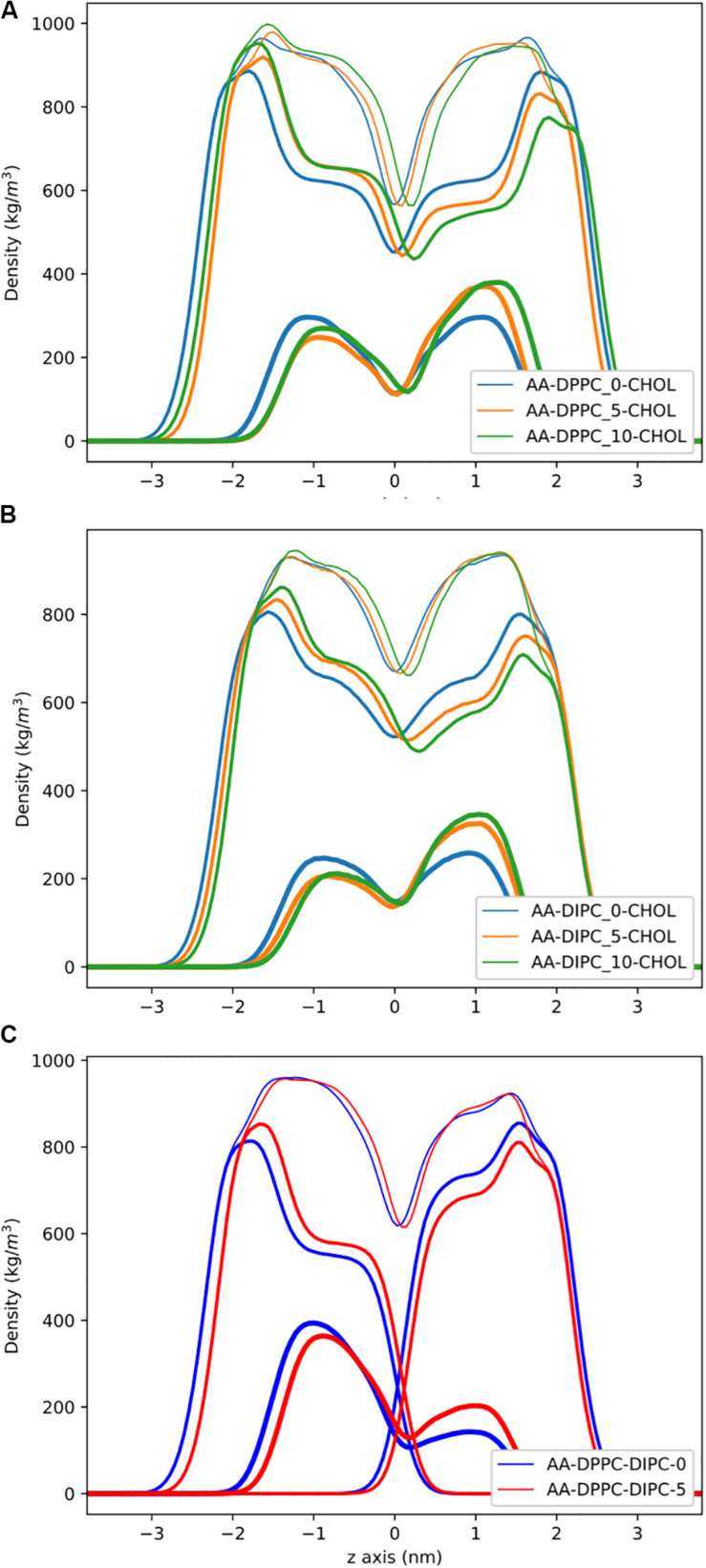
Partial density profiles for the AA lipid bilayers with 30 mol% cholesterol. **(A)** Profiles for DPPC. **(B)** Partial densities for DIPC. **(C)** Bilayers with asymmetric DPPC in one leaflet and DIPC in the other. Thin lines are for the whole bilayer, medium lines are the PC lipids, and cholesterol is thick lines.

Partial density profiles for the AA bilayer with cholesterol are shown in Figure 6. Similar to the CG results, we observe substantial differences in the PC lipid density, by removing lipids in one leaflet, but the cholesterol density compensates, and the overall bilayer density has little change. The DIPC(upper)/DPPC(lower) bilayer with cholesterol, shows a substantial concentration of cholesterol in the lower DPPC bilayer, causing a large difference in the overall bilayers density between leaflets. This matches the results from the CG model. The DIPC(upper)/DPPC(lower) bilayer with five fewer lipids in the DIPC bilayer has a similar trend as the pure PC membranes, with cholesterol compensating for asymmetric PC density. This results in very similar overall bilayer densities for the DIPC(upper)/DPPC(lower) bilayer with and without equal number of lipids in either leaflet. The APL for the AA cholesterol membranes (Table 2) have the same trends as the CG membranes, with little changes in area when lipids are removed from only one leaflet.

**FIGURE 7:**
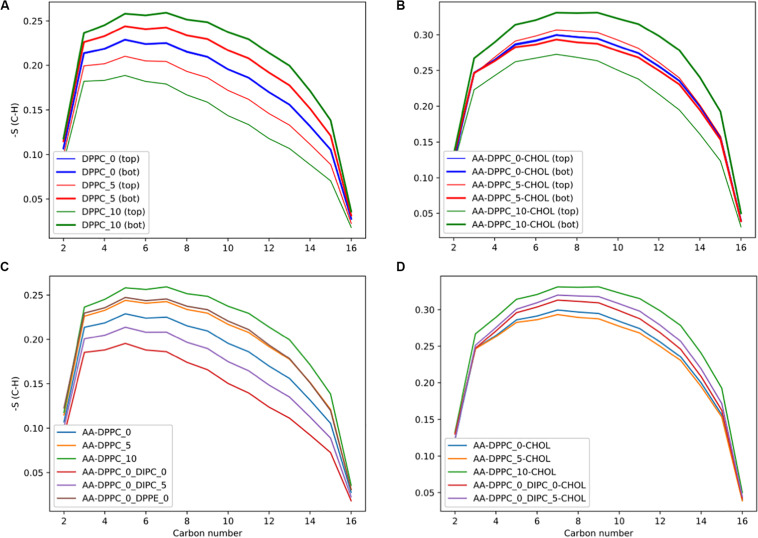
DPPC lipid chain order parameters for the DPPC’s *sn-*2 chain. **(A)** Order parameters for the pure DPPC bilayer, comparing the top and bottom leaflets, and **(B)** with 30 mol% cholesterol. **(C)** Comparing order parameters for the top leaflet in the different membranes with compositional asymmetry, and **(D)** with 30 mol% cholesterol.

To assess the effect of asymmetry on the individual lipid’s structures, we calculated the lipid tail’s order parameter. The order parameter is a measure of the membranes ordering, based on the lipid tails alignment with the membrane normal. A value of 0 indicates no ordering and 1 completely ordered. Figure 7A shows that the bilayers compensate for the change in number of lipids by the leaflet with more lipids becoming more ordered, and the leaflet with fewer lipids becoming less ordered. Figure 7B shows that the DPPC lipids are more ordered with cholesterol, and the effect of asymmetry is reduced. For the 5 DPPC lipid difference, we observe the opposite trend as the other membranes, with the leaflet with more lipids having a slightly lower order parameter, and the one with fewer lipids more ordered. This could be due to an artifact of having the wrong distribution of cholesterol from the starting CG structure, although the difference is quite small. Future work explicitly studying cholesterol’s chemical potential would be of interest to properly assess this discrepancy.

We calculated the order parameter for DPPC lipids in the mixed composition membranes (Figure 7C). In the DIPC(upper)/DPPC(lower) membrane the DPPC molecules have less order, as they are forced to compensate for the more disordered lipids in the opposite leaflet. We also tested the effect of asymmetric membranes with different headgroups, with DPPC in one leaflet and DPPE in the other. The DPPC in the DPPE(upper)/DPPC(lower) bilayer had high order, with a very similar profile as the DPPC_5 upper leaflet. This illustrates how the leaflets can compensate for the other, with the DPPC becoming more ordered to accommodate the DPPE in the opposite leaflet, with a much lower APL. With 30 mol% cholesterol (Figure 7D), there are interesting differences, with the DPPC lipids in the DIPC(upper)/DPPC(lower) mixed membranes having a higher order parameter compared to the pure DPPC bilayer. This is due to the increased concentration of cholesterol in the DPPC leaflet.

**FIGURE 8:**
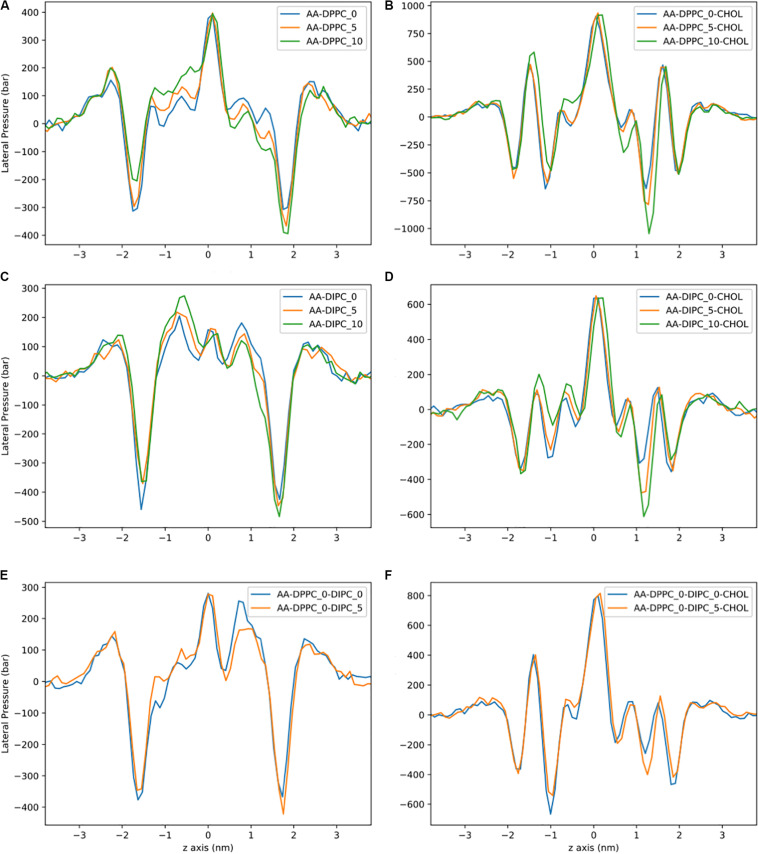
Lateral pressure profiles for AA bilayers with asymmetric number of lipids between leaflets. **(A)** DPPC. **(B)** DPPC:CHOL. **(C)** DIPC. **(D)** DIPC:CHOL. **(E)** DIPC(upper)/DPPC(lower). **(F)** DIPC(upper)/DPPC(lower):CHOL.

The LPPs for the AA bilayers are shown in Figure 8. These pressure profiles have large local pressures of hundreds of bars. For the pure DPPC bilayers, there is a very large trough in the pressure at the headgroup region, and a large positive peak at the bilayer center. Removing lipids from one leaflet has an effect on the depth of the trough at the DPPC headgroup region and no effect on the height of the peak at the bilayer center. Similar to the CG model, the biggest change in the pressure profile is observed in the lipid tail region of the DPPC bilayers. Similar behavior is observed for the pure DIPC bilayers (Figure 8B), but with less of an effect compared to DPPC. The bilayers with 30 mol% cholesterol have qualitatively different pressure profiles, with larger pressures, and a second headgroup trough and headgroup peak, compared to the bilayers without cholesterol. This behavior has been observed in other simulations of cholesterol (Shahane et al., 2019). The cholesterol bilayers show a substantial shift in the headgroup region for the asymmetric bilayers. There is also no effect on the magnitude of the pressure peak at the membrane center for asymmetric bilayers.

### Ion and Electrostatic Imbalances

**FIGURE 9:**
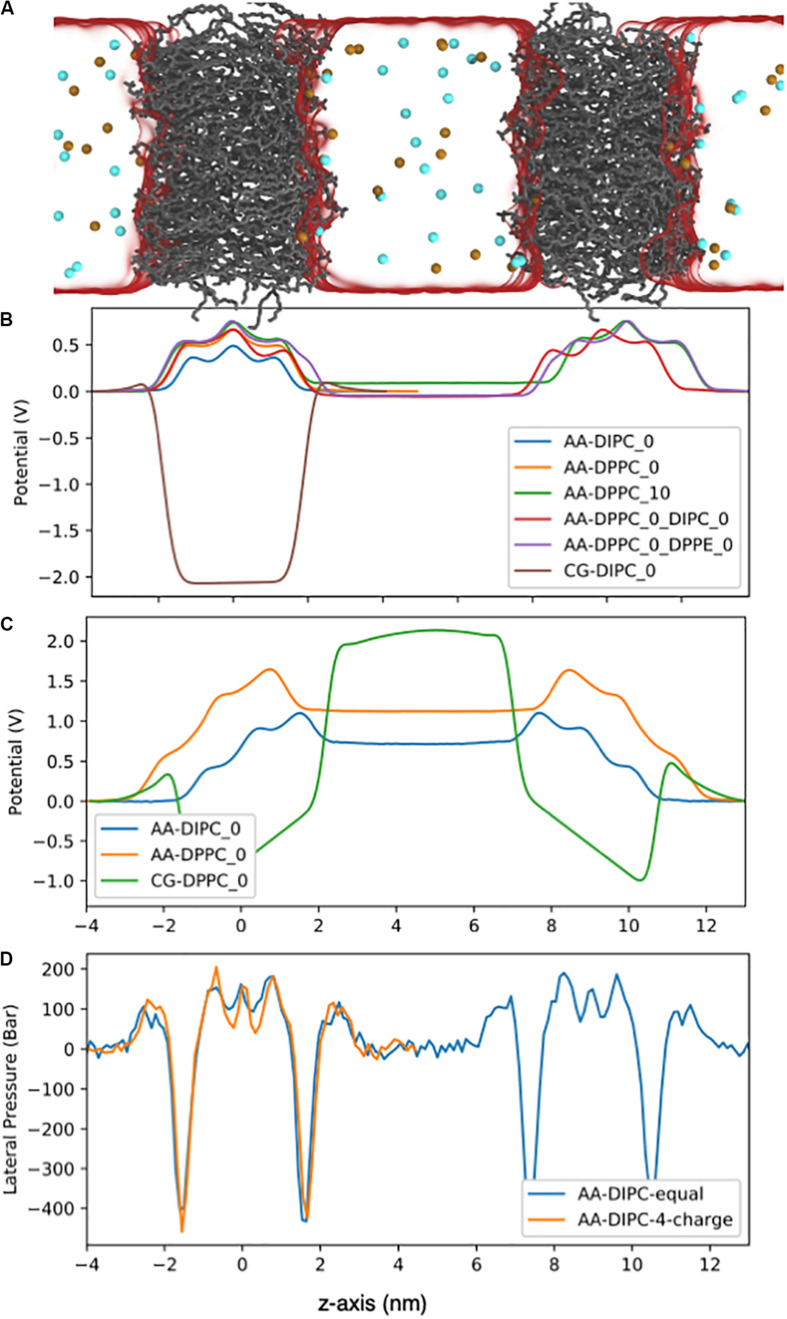
Electrostatic potential for the AA lipid membranes. **(A)** Snapshot of the double bilayer set-up. The DPPC lipids are shown as gray lines, ions are red and blue balls, and water is a translucent surface. **(B)** Bilayers with no charge imbalance between aqueous compartments. The potentials were set to zero on the left side of the bilayer. **(C)** Double bilayer systems with a 4e charge imbalance. Water regions have a flat potential due to conducting counter ions. **(D)** Lateral pressure profile for the DIPC double bilayer with and without a 4e charge imbalance.

The membranes electrostatic potential can have important consequences on many biological functions of membranes and membrane proteins. Figure 9A shows the system set-up and the electrostatic potential (Figure 9B) across lipid membranes that do not have a charge imbalance. The bilayers have a relatively large positive potential at the bilayer center. The strength of the potential depends on the bilayer structure and composition, with a lower potential for the more disordered, unsaturated bilayers. For symmetric bilayers, the potential is equal to zero on both sides of the membrane. The poly unsaturated DIPC bilayer has a lower potential at the bilayer center compared to the DPPC bilayer. Asymmetric membranes cause the potential to shift between the two aqueous environments, even though both contain 150 mM NaCl, and there is no charge imbalance. Reducing the number of lipids on one side (DPPC_10) causes an increase in potential of ∼100 mV, while having DIPC or DPPE on the other leaflet causes a potential of ∼-70 mV. We also show the electrostatic potential across a Martini DIPC bilayer (Figure 9B). The Martini model does not reproduce the positive potential at the membrane center, which is a well-known short coming of the model.

Another source of membrane asymmetry is imbalances in the composition of the aqueous phases that the bilayer separates. Many biological membranes have an electrostatic potential across the bilayer. To investigate this effect, we simulated lipid bilayers with a net charge imbalance (4e) across the membrane. Figure 9C shows the electrostatic potential across pure DPPC and pure DIPC lipid bilayers with a 4e charge imbalance. The charge imbalance causes a potential difference of ∼1.2 V across the DPPC bilayer and ∼0.75 V across the DIPC bilayer. This illustrates the importance of the bilayer structure on the electrostatic potential gradients. The relatively large charge imbalance has a small effect on the bilayer structure, as shown by the lateral pressure profile in Figure 9D. The APL for DPPC increases to 0.63 from 0.62 nm^2^ without a charge imbalance. Supplementary Figure S6 shows the order parameters for the 4e DPPC bilayer are slightly reduced compared to the pure DPPC bilayer. This modest change in structure is somewhat surprising, given that if we simulated long enough, a pore would likely form across the membrane, to allow the ions to equilibrate between water compartments.

## Discussion

Biological membranes contain incredible complexity and diversity. While it has long been known that biological membranes contain asymmetry between one side and the other, much remains unknown about the origin, physical effect, biological function, and consequences of its disruption. Here we have investigated a number of different types of membrane asymmetry using both atomistic and CG models. Our results illustrate how lipid membranes are complex and cooperative aggregates. Changes on one side of the membrane affect the opposite leaflet. In general, the two leaflets attempt to balance asymmetric deviations. For example, if one leaflet becomes more ordered, the opposite leaflet will become more ordered. How the bilayer responds to the asymmetry depends on the composition and chemical structure of its lipids. We chose simulation boxes that were small, to preclude the effect of bilayer bending in response to asymmetry. Future work on the system size dependence of asymmetry would be of interest, or large-scale CG simulations of small unilamellar vesicles with asymmetric lipid distributions.

Here we have used both atomistic and CG simulations to study a number of asymmetric bilayer systems. While there are many detailed insights, the overall trend we observe is the coupling between the bilayers two leaflets. By running extensive simulations for CG systems with 3–4 lipid components and systematically changing the asymmetry, we show that the number lipids in each leaflet can be adjusted to minimize leaflet ST (Figures 1C,F). The resulting number asymmetry is somewhat higher than using estimates from the APL of the two symmetric bilayers. This effect had been shown previously (Doktorova and Weinstein, 2018; Hossein and Deserno, 2020), but here we have run extensive CG simulations to characterize the behavior with and without cholesterol. Hossein and Deserno (2020) also showed that in some cases a more complex balance between spontaneous curvature and tension is desired. Because the two leaflets are interacting, they will alter the others elastic properties and therefore shift the packing of the lipids. While this can have important consequences, we also note that many bilayer properties are quite similar over a wide range of asymmetries (Figure 1D), suggesting that getting the exact number asymmetry may not seriously impact many simulation results. This effect is also due to the lipid’s flexibility, where each leaflet compensates, so the difference between the two leaflets is minimized.

We have also addressed the influence of cholesterol on membrane asymmetry. Cholesterol has been hypothesized to be able to reduce membrane asymmetry by being able to flip quickly from one leaflet to the other and equilibrate membrane stress (Miettinen and Lipowsky, 2019). Our results suggest that for the systems tested here this is true for some properties, such as the overall membrane density and APL, cholesterol can also increase the effect on some other properties, such as the LPP. Cholesterol makes the situation of simulating membrane asymmetry more complicated, because it is able to flip-flop on a time scale easily accessible to CG simulations, but not for AA simulations of long saturated lipids. This is also likely the case for other fast flipping lipids such as fatty acids, ceramides and diacylglycerol (Bennett and Tieleman, 2012; Janke et al., 2014). Cholesterol’s distribution is also more complicated because it will be driven by its chemical potential for either leaflet, and its ability to influence the membrane’s spontaneous curvature (Allender et al., 2019; Hossein and Deserno, 2020). Considerable work in this area is needed to properly assess lipid distributions, such as free energy methods for determining their relative chemical potential in each leaflet.

By systematically investigating AA and CG models, we are able to compare specific difference for the effect of membrane asymmetry. We find that many of the properties and differences are captured using both levels of detail. While there are substantial differences in the shape and magnitude of the LPPs, many of the changes for asymmetric membranes are similar for the two models. This is crucial for future simulation work using multiscale approaches to study asymmetric membranes. These methods are attractive due to the fast sampling of the CG models, which is necessary for many slow processes involving lipids, and the fine chemical details provided by the AA models. Ensuring that the CG asymmetric lipid distribution matches the AA models is necessary, or the AA simulation will be biased by the initial CG simulation result. The electrostatic potentials for Martini are shown to be opposite to that of AA models, illustrating a fundamental limitation for the model. It is also likely that the large preference of cholesterol for DPPC over DIPC in the asymmetric bilayer could be due to Martini’s strong repulsion between cholesterol and poly unsaturated phospholipids. Future work comparing the lipid chemical potentials for both models might help provide more qualitative differences. The overall good agreement between the AA and CG results is promising for large scale multiscale membrane simulations. Future work investigating the effect of proteins inclusion in asymmetric membrane systems would also be of interest. Another aspect of asymmetry for future work are pH gradients, which require more sophisticated computational methods.

We examined how asymmetric ion distributions can affect the membrane structure, as well as how asymmetric lipid distributions influence the electrostatic potential across the membrane. Our results match previous results showing that changes in lipid headgroup can shift the overall membrane electrostatic potential (Gurtovenko and Vattulainen, 2007). We also show that changing the lipid tails can have a substantial effect on the membrane’s dipole potential. The fact that lipid distribution influences membrane electrostatic potentials has important implications on numerous biological processes, such as nerve cell activation. Another source of membrane asymmetry is the bilayer separating aqueous compartments with chemical and/or ionic gradients. We show that relatively large electrostatic potentials have a modest effect on the membranes overall structure. This is somewhat surprising, as these membranes are likely to form pores if simulated for long times, so the ions can equilibrate (Gurtovenko and Vattulainen, 2005). These large electrostatic gradients might promote structural fluctuations that promote pore formation, such as water wires across the membrane, which have been shown to be a pre-pore state for pore formation in lipid bilayers (Bennett et al., 2014). Our results also illustrate how changes in lipid tails have a large effect on the electrostatic potential resulting from ionic imbalances. These differences are important for many biological processes, including the activity of voltage-gated ion channels (Bezanilla, 2007), GPCR signaling (Rinne et al., 2015) and the insertion and penetration of charged peptides, such as antimicrobial peptides (Teixeira et al., 2012) and cell-penetrating peptides (Moghal et al., 2020).

## Conclusion

Asymmetry in lipid bilayer simulations is a crucial form of complexity in biological systems. Cells create and exploit asymmetry for energy production, signaling, and general transport of molecules. Studying membrane asymmetry in model systems is challenging for experiments and simulations, as this is an inherently non-equilibrium situation. For simulations, finite system sizes and time scales makes membrane asymmetry hard to study. Our results show how changes in number of lipids, lipid headgroups, lipid tails, ionic imbalances, and the effect of cholesterol impact bilayer’s structural properties. Overall, we show that CG Martini simulations reproduce the effects observed with AA CHARMM36 simulations. One leaflets structure effects the opposite leaflet, as the individual lipids adapt to their local environment. Fast flip-flop of cholesterol creates additional challenges, for simulating asymmetric membrane simulations. Cholesterol reduces the impact of membrane asymmetry for some structural properties, but can create more imbalance, especially when it has a large preference for lipids in one leaflet over the other. There is much work in the future for membrane simulations of asymmetry, including assessing chemical potential differences and enhanced sampling methods.

## Data Availability Statement

The datasets generated for this study are available on request to the corresponding author.

## Author Contributions

MB, SH, ML, LM, and MU wrote the analysis scripts. PS and HI managed the project. All authors contributed to running simulations, analyzing the result, and conceiving the project.

## Conflict of Interest

The authors declare that the research was conducted in the absence of any commercial or financial relationships that could be construed as a potential conflict of interest.

## Footnotes

1 https://github.com/llnl-hmc-clinic/Bilayer-Simulation-Pipeline
