# Supplementary material for: Simulations of Asymmetric Membranes Illustrate Cooperative Leaflet Coupling and Lipid Adaptability
Source: Front Cell Dev Biol. 2020 Jul 21;8:575. doi: 10.3389/fcell.2020.00575 (PMC7396604; doi:10.3389/fcell.2020.00575)
Supplement: Supplementary file 1 [file Image_1.pdf]

Supplementary Figures

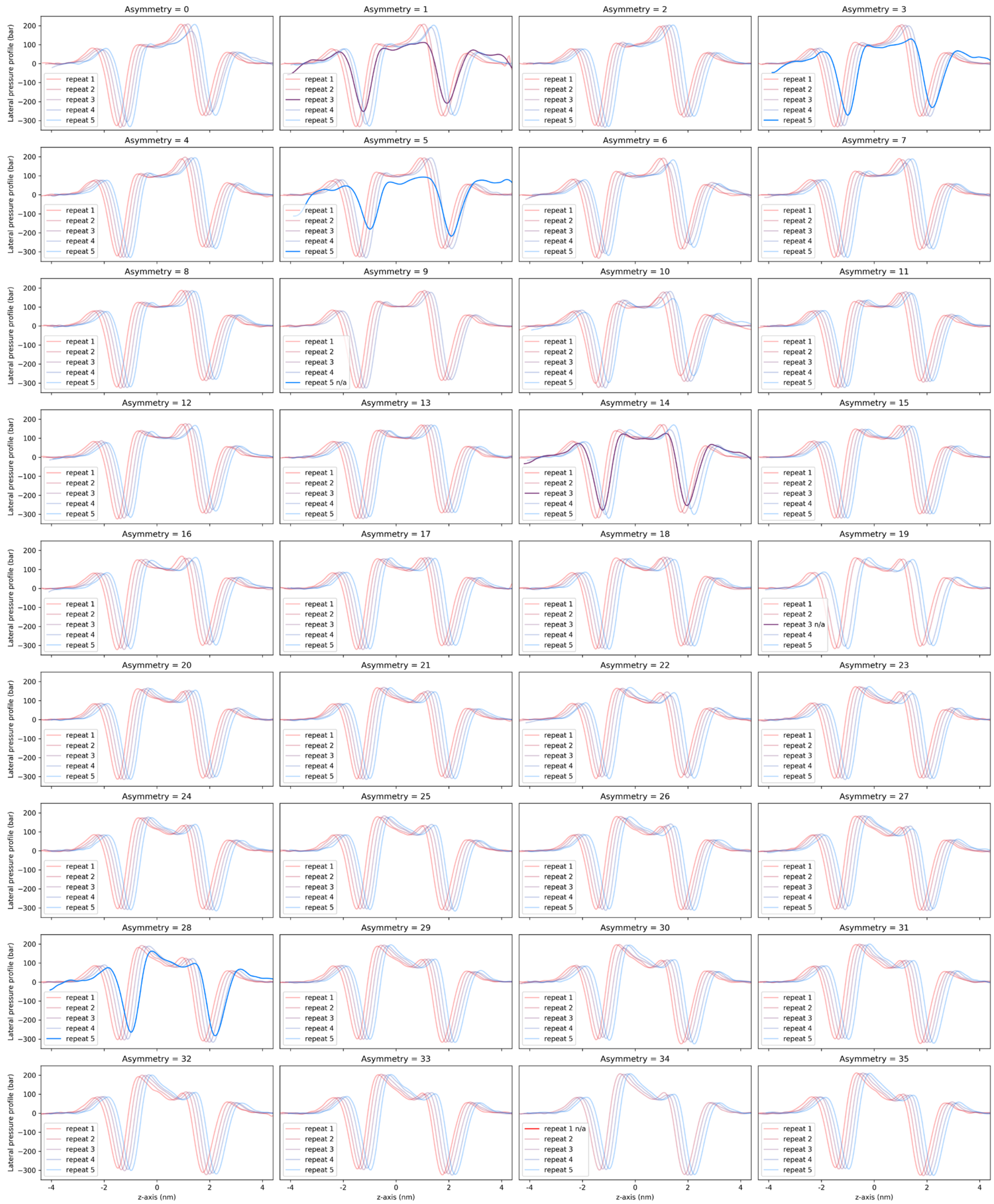

Figure S1: The individual lateral pressure profile (LPP) plots are shown for all 180 simulations for bilayers without cholesterol. Each of the 36 systems of differing degrees of asymmetry had five repeats (colored red to blue). Each simulation was run for 10 µs, with the last 2 µs used for analysis. Simulations that crashed or failed are noted with ‘n/a’. Other simulations where the profile fails to adopt the distinctive typical features of an LPP plot are also highlighted. These

highlighted outliers are excluded from the data when generating the average LPP plots show in Fig. 1E. For visual clarity, each repeat is plotted 0.125 nm further along the x-axis.

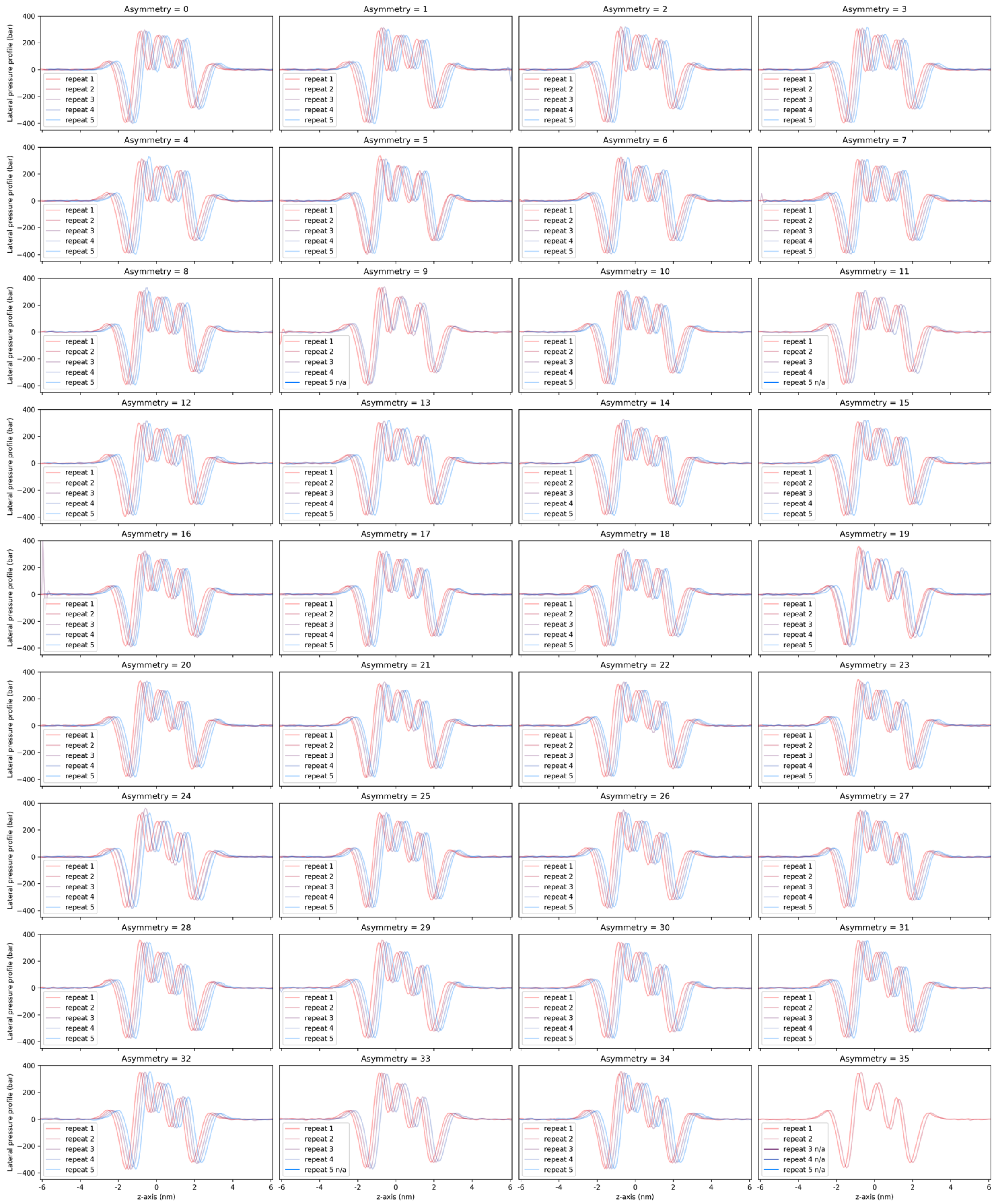

Figure S2: The individual lateral pressure profile (LPP) plots are shown for all 180 simulations for bilayers with cholesterol. Each of the 36 systems of differing degrees of asymmetry had five repeats (colored red to blue). Each simulation was run for 10 μs, with the last 2 μs used for analysis. Simulations that crashed or failed are noted with ‘n/a’. Other simulations where the

profile fails to adopt the distinctive typical features of an LPP plot are also highlighted. These highlighted outliers are excluded from the data when generating the average LPP plots show in Fig. 1E. For visual clarity, each repeat is plotted 0.125 nm further along the x-axis.

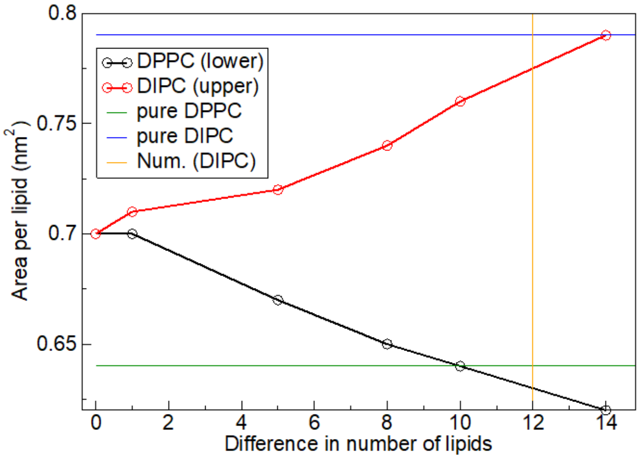

Figure S3: The resulting change in APL in the DIPC(upper)/DPPC(lower) bilayers from the change in number of lipids in the upper DIPC leaflet. The number of DIPC is calculated assuming 64 DPPC molecules in the lower leaflet. The APL for the pure DPPC and DIPC bilayers are plotted for reference as well as the asymmetry chosen from matching APL from the symmetric simulations (yellow line).

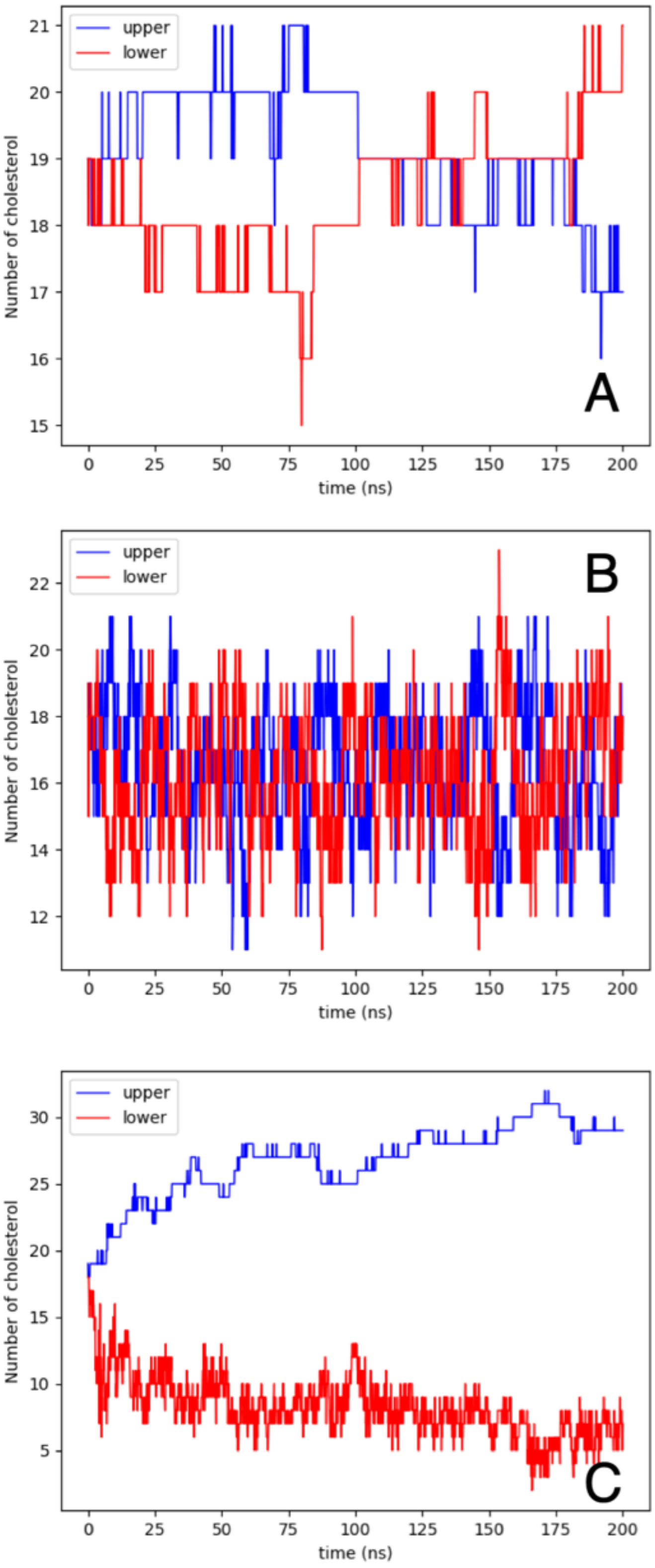
upper
lower
Number of cholesterol
time (ns)
A
B
C

Figure S4: The number of cholesterol molecules in each CG bilayer leaflet. The simulations were started with 19 cholesterols in each leaflet (30 mol%) (**A**) DPPC. (**B**) DIPC. **(C)** the asymmetric DIPC(upper)(blue line)/DPPC(lower) (red line) membrane.

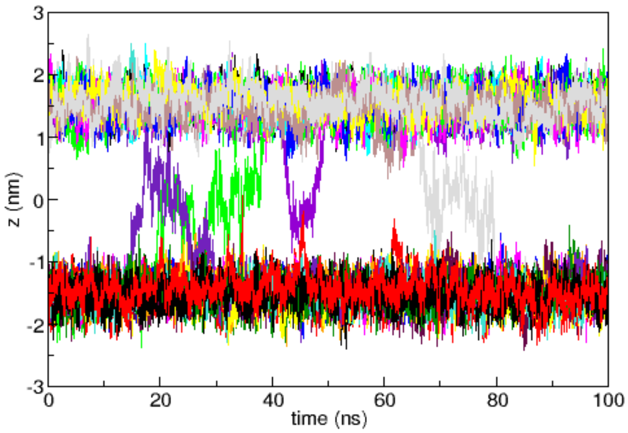

Figure S5: Z-position for the head groups of the 38 cholesterol molecules in the DIPC-30mol% cholesterol bilayer. There are 2 flip-flop events and 2 other near flips during the 100 ns of simulation data.

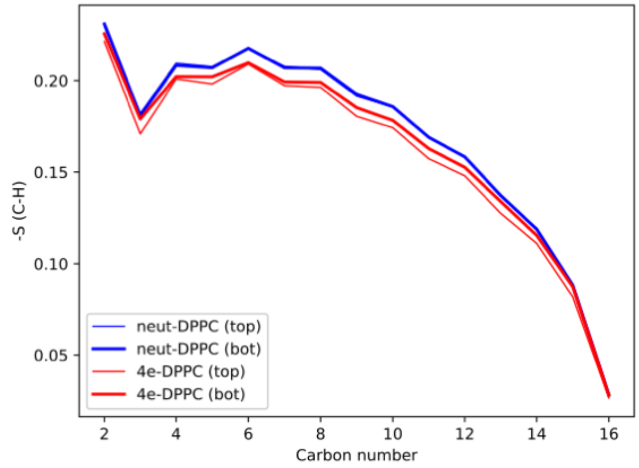

Figure S6: Order parameters for the DPPC lipid chains in pure DPPC membranes with and without a charge imbalance.
